# Monthly Summary

**Published:** 1889-08

**Authors:** 


### Monthly Summary.

Anæsthetics in Dental Practice.—In *The Journal* of March we noticed editorially a case of death from chloroform in a dentist's chair. Dr. J. C. Reeve, of Dayton, Ohio, has kindly sent us the October, 1888, number of the *Dental Register*, containing an article on "Anæsthetics in Dental Practice" from his pen, which, on the subject of anæsthetics, is one of the ablest in the profession. Dr. Reeve says:—"There is no professional duty I perform so unwillingly as that of admisistering an anæsthetic for dental purposes, no fee that I consider so hardly earned as that which I receive for this service. At the same time I am frequently giving anæsthetics for general surgical purposes without hesitation and without undue anxiety." Again he says: "Are anæsthetics more dangerous in dental practice than in general surgery? The answer must be unqualifiedly in the affirmative. Without attempting to collect statistics take only those of the Royal Medico-Chirurgical Society and those of Sansom. The one gives 8 cases of death under tooth-drawing out of 100 of all operations, and the other 12 out of 107. Here then is nearly 10 per cent. of all the deaths occuring in dental operations. But this statement alone gives no just idea of the relative mortality. This could only be accurately ascertained if the total number of administrations in all surgical operations was known. Certainly anæsthetics are administered for general surgical purposes hundreds of times for once in dental practice, and if so, then the relative number of deaths under tooth-drawing is enormously large. The causes of the high rate of mortality during this particular operation are not far to seek. I do not believe that the entrance of blood into the air-passage is very important. Several deaths, however, have been caused by an extracted tooth falling into the larynx, without doubt due to the position of the patient. Anæsthetics should never be administered unless the patient be recumbent. This is not, however, in my opinion, a very potent factor, and was fully considered in the paper. Another is the particular nerve involved in the dental operation, the acute pain caused by injuries to it, and the powerful effect of sudden impressions upon its branches upon the great and vital processes of respiration and circulation. By sudden impressions upon this nerve more than any other, is that inhibition of the heart's action brought about which is sudden death. Far more important than all, however, is the fact that the induction of anæsthesia for tooth-drawing is likely to be incomplete, and will pretty certainly be so if the operator is also the administrator. Now it is a positive doctrine of the highest and latest authorities that such reflex actions as above given are increased under chloroform, that a state of partial anæthesia is therefore one of especial danger, one especially so if the pain produced is at once sudden and sharp. It is gratifying, therefore, to see that this source of danger is fully recognized by the author of the paper, although it is not emphasized as it deserves to be. There is no more seductive procedure than to give a few whiffs of chloroform for the extraction of a tooth; there is no more dangerous practice. If an anæsthetic is given at all, it should be given until the patient is 'off.'. There is no plainer doctrine than this connected with the subject."

Dr. Reeve wholly dissents from the doctrine that a full dose of whiskey before the administration of the anæsthetic secures safety. There are on record many cases of death from chloroform in which an alcoholic stimulant was given just before the fatal inhalation. In regard to bromide of ethyl, Dr. Reeve thinks it is a dangerous agent, on account of its bad record, and its marked perturbative action on the heart. He does not know of such objections to the use of nitrous oxide as will justify dentists in resorting to stronger anæsthetics. The objections adduced, he says, "seem but trivial when the tremendous responsibility is considered which the dentist takes upon himself when he proceeds to administer chloroform or ether, when the awful calamity of a sudden death from these agents comes to mind."

It may be said, finally, that when a dentist administers chloroform for the purpose of pulling a tooth, he incurs a responsibility that he has no right io incur.—*Journal of the American Medical Association, Chicago.*

Cold Water in Facial Neuralgia.—Dr. E. Orvin Barker writes: What I have to say on this subject will perhaps not be new to many readers of the *Brief*, while to very many it will no doubt be.

Some two years ago I was called in to see a patient who was being treated by Dr. G., of this place, for facial neuralgia. The doctor being absent at the time, I found the patient—a large, muscular man, about thirty years of age—suffering excruciating pain in one side of the face, with eye almost swollen shut. I gave him a large dose of sulph. morphia, hypodermically, assured him that it would relieve him, and left. Perhaps two hours after, I met Dr. G. and told him what I had done and that perhaps he had better call and see the man. He requested me to stop with him, and on going in we found patient suffering very much, and the family applying hot applications to the face, which they had been doing from the beginning of the attack.

Dr. G. had them remove the hot applications and replace them with cold; and in one minute the patient was easy and had no more trouble in controlling the attack.

Some time after this I was called one morning to see a Mr. H., a large stout man of about fifty, and found him suffering great pain in one side of the face, and his wife constantly applying hot applications. Remembering the case above referred to, I ordered the hot applications removed and replaced by cold, and almost immediately the whole muscular system seemed to relax, and the patient cried out that he was gone, that he was dying, and called his wife and son to him and bade them good-bye. Of course, they were greatly frightened, and I was accused of killing him. But a glance at the patient was sufficient to tell me that he was not dying. However, I told them to remove the cold cloth and in a very few seconds he was painfully reminded that he was not a corpse by any means. A hypodermic injection of morphia sulph. soon relieved the pain.

Not long ago I was called to see a lady, perhaps thirty years of age, who was subject to, and suffering at the time with a severe attack of the same trouble, and was treating it with hot applications. This patient thought that she could not take morphia, so I concluded to try the effect of cold water. Accordingly, I applied a cloth wet with cold water, and, very soon the patient remarked that she felt so queer, and then cried out that she was sinking, and called her husband to her and seemed to think that she was actually dying. As in the other case the cloth was removed and the pain soon returned, and had to be relieved by the use of other remedies.

Now, what was the cause of the strange feelings? Was it all owing to the shock to the nervous system, caused by the sudden change from hot to cold? Or was it partially due to a sort of an ecstasy resulting from the instant relief from such great pain?

I am inclined to think that in both cases, as did it in the first, it would have resulted in a cure had the cold been retained.

Have any of the brethren had a similar experience?

No one has ventured to give any cause for the absence of the usual amount of blood in the case of bloodless labor reported some time ago.—*Med. Brief.*

---

FAULTS OF COPPER AMALGAM.—In 1886* I described a new method for making dental amalgams by electric deposition. In this paper particular attention was called to the value of this new method for producing copper amalgam, the mass so produced being perfectly free from the presence of other metals; and moreover it contained no copper oxides or sulphides, and on this account was stronger when hardened.

This new method has led to the somewhat extensive introduction of copper amalgam. I wish this account to give the results of several years' observation on this material when used in filling teeth. I was led to its extensive use in my practice through the statements of several eminent men, who said that it was free from shrinkage, did not change its shape, and did not discolor the teeth, when pure. My experience shows that all these statements are false. In mouths in which decay is not rapid, copper amalgam turns dark from the formation of a

*Boston Medical and Surgical Journal, page 175, 1886.

sulphide, and preserves the teeth as well as the average gold filling. In such mouths the teeth are usually of good structure, and are not stained by the filling unless they are pulpless, in which case discoloration of the substance of the dentine is sure to be simply a matter of time. Discoloration of the surfaces of the teeth is always present to a greater or less degree, however.

In mouths in which the saliva is still more abnormal, and in consequence of which the teeth decay rapidly, the amalgam does not turn black as a rule, at least there are periods in which the surface is either wholly or partly clean. I have seen copper amalgam fillings lose half their weight in such mouths in two years' time. A loss of five per cent. a year is common. In cases of rapid loss of substance the surface of the fillings show a fine crystalline appearance not unlike that of the fresh fracture of high-grade steel. In such cases it cannot be denied that as a preserver of the teeth from caries the filling is of value; but until it can be shown that the continual introduction of so much copper and mercury into the system is harmless I advise caution in the use of copper amalgam.

Finally, copper amalgam changes its shape after it is introduced into the tooth, and therefore does not make a tight filling. This is so contrary to all that has been said on this subject that I am prepared to have the statement doubted, but nevertheless it is a fact.

It is best to test the matter in a simple way first, after which careful tests of the specific gravity can be made to prove it. I recommend using large amounts (20 grammes) of the filling, and packing it into large glass tubes, which are kept at a temperature not varying widely from that of the mouth. It will be seen that the first appearance of leakage is greatest near the surface of the plug, and that as the change in shape progresses the ink penetrates deeper and deeper between the glass and the fillings; indeed, the action of the fluid is such as to suggest the truth of the statement about the spheroidal tendency of amalgams.— *William Herbert Rollins, in the Dental Cosmos.*

IMPURE DRUGS.—The report of Willis G. Tucker, M.D., Ph. D., Analyst of Drugs to the New York State Board of Health, is both suggestive and instructive. It contains the results of nine months of work, during which time three hundred and twenty-six samples of drugs and pharmaceutical chemicals and preparations have been collected and examined. Fifty-three samples of purified chloroform were examined; thirty-nine of these were of good quality, ten were fair, and four inferior. The same number of samples of stronger ether was examined; twenty were of good quality, five were fair, twenty-six were inferior, and two were not as called for, one of them being spirits of nitrous ether, and the other the so called "Concentrated Nitrous Ether." The former of these was dispensed unlabeled, and the second was labeled "U. S. P. Stronger Ether" by the seller. In commenting on the result of the ether examination, Dr. Tucker well says:

"Such errors as these are the grossest of blunders, and the consequence of such ignorant or careless sales might be most serious to the purchaser. Stronger ether being generally used as an anæsthetic ought to be of good quality; but from these results it will be seen that an inferior article is often, indeed in the majority of cases, offered for sale. Stronger ether having been called for in writing in each case, the substitution of common ether is entirely inexcusable and shows great carelessness or ignorance."

It is gratifying to learn that such excellent work is being done by our State Board and that it is proposed to continue it. Dr. Tucker states that the work done during the past two years has had a decided effect in improving the quality of the drugs sold throughout the State, and expresses the opinion that if manufacturers, dealers and others interested will co-operate with the Board in the efforts it is making to raise the standard of the drugs and medicinal preparations now upon the market, the desired end can be attained without interfering with any legitimate business industry, or embarrassing dealers by a too rigid or oppressive enforcement of the law.—*Brooklyn Medical Journal.*

